# Supplementary material for: Conditional chemoconnectomics (cCCTomics) as a strategy for efficient and conditional targeting of chemical transmission
Source: eLife. 2024 Apr 30;12:RP91927. doi: 10.7554/eLife.91927 (PMC11060718; doi:10.7554/eLife.91927)
Supplement: Figure 3—source data 3. [file elife-91927-fig3-data3.zip › Figure 3 source data3 part 2.zip!/Figure 3-Source data 3-part2/1053-1-CRISPResso_Report_mpeqg9da.zip!/CRISPResso_on_1053_3/1a.Read_barplot.pdf]

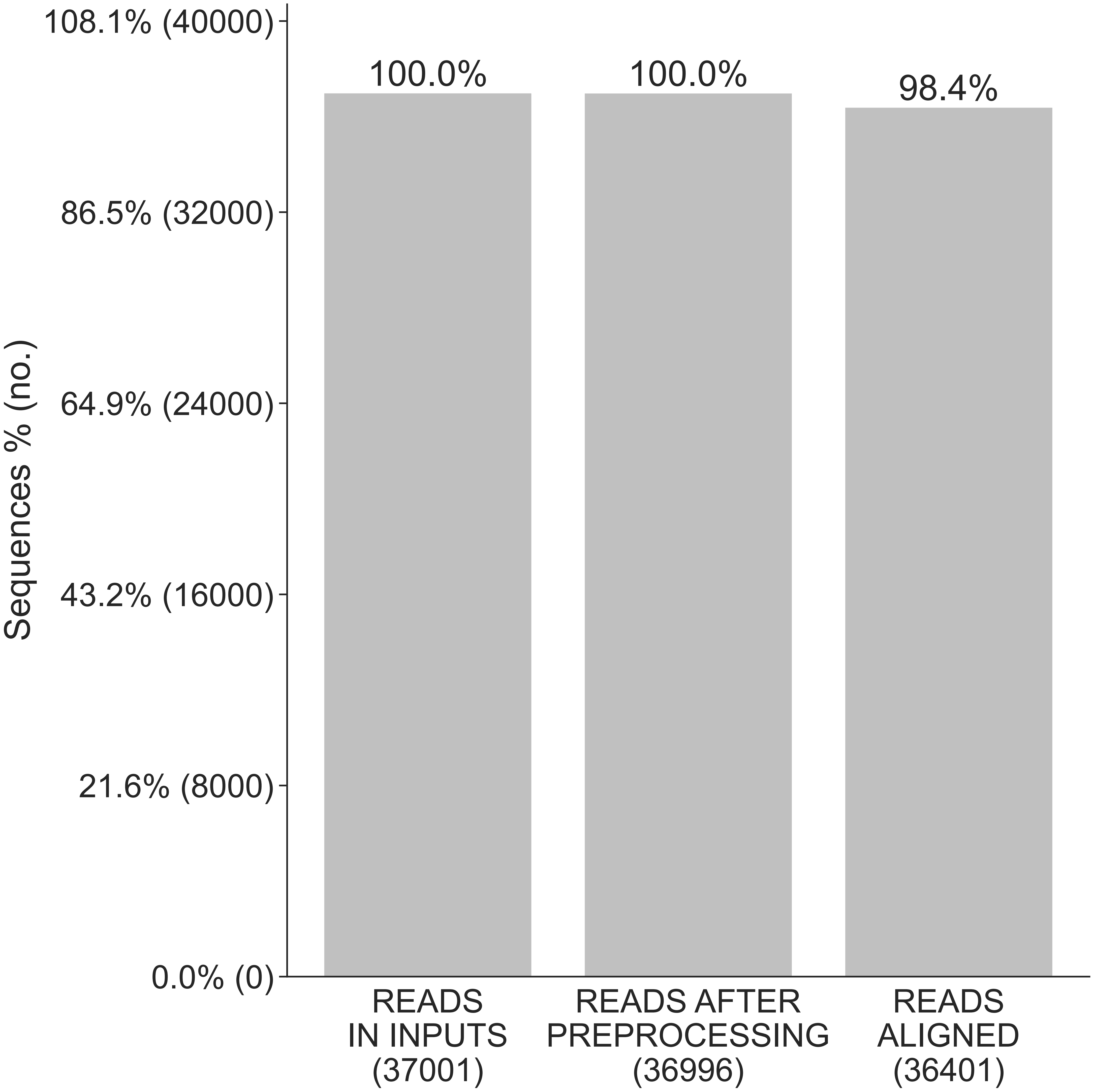

Sequences % (no.)
108.1% (40000)
86.5% (32000)
64.9% (24000)
43.2% (16000)
21.6% (8000)
0.0% (0)
100.0%
100.0%
98.4%
READS IN INPUTS (37001)
READS AFTER PREPROCESSING (36996)
READS ALIGNED (36401)
